# Multimodal Imaging-Based Cerebral Blood Flow Prediction Model Development in Simulated Microgravity

**DOI:** 10.34133/cbsystems.0448

**Published:** 2025-11-24

**Authors:** Linkun Cai, Yawen Liu, Kai Li, Changyang Xing, Zi Xu, Lianbi Zhao, Ke Lv, Zhili Li, Hao Wang, Linjie Wang, Dehong Luo, Lijun Yuan, Lina Qu, Yinghui Li, Zhenchang Wang, Pengling Ren

**Affiliations:** ^1^ School of Biological Science and Medical Engineering, Beihang University, Beijing 100191, China.; ^2^ Department of Radiology, Beijing Friendship Hospital, Capital Medical University, Beijing 100050, China.; ^3^ State Key Laboratory of Space Medicine, China Astronaut Research and Training Center, Beijing 100094, China.; ^4^ Department of Ultrasound Medicine, Tangdu Hospital, Air Force Medical University, Xi’an 710038, China.; ^5^ Shenzhen Center, Cancer Hospital Chinese Academy of Medical Sciences, Shenzhen 518116, China.

## Abstract

**Background:** Abnormal alterations in cerebral blood flow (CBF) have been implicated in cognitive decline and neurodegeneration. Maintaining adequate CBF in astronauts during long-duration microgravity is therefore crucial for the success of manned spaceflight. However, the quantitative assessment of CBF during space missions remains challenging. **Methods:** Thirty-six participants underwent a 90-d −6° head-down tilt bed rest (HDTBR) protocol, a well-established ground-based analog of microgravity. Multimodal imaging data, including internal carotid artery Doppler ultrasound and brain magnetic resonance imaging, were collected during HDTBR. Multiple machine learning (ML) algorithms were developed to investigate carotid–CBF mapping relationship and establish CBF change prediction models. **Results:** After 90-d HDTBR, significant regional CBF decreases were observed, primarily in the right Heschl’s gyrus, right middle cingulate gyrus, and right superior frontal gyrus. The optimal ML model CatBoost showed robust predictive performance for CBF in these regions (right Heschl’s gyrus: AUC = 0.88, accuracy = 0.84; right middle cingulate gyrus: AUC = 0.92, accuracy = 0.83; right superior frontal gyrus: AUC = 0.82, accuracy = 0.72). To enhance accessibility and practical utility, the prediction model was implemented as an interactive web application for in-orbit deployment. **Conclusion:** This study demonstrates the feasibility of constructing ML-driven CBF prediction models under microgravity based on multimodal imaging. The developed prediction models show promise as early warning tools for brain health of astronauts in spaceflight.

## Introduction

During spaceflight, alterations in gravity and posture cause fluid shifts within the body. The head is particularly susceptible to these fluid shifts due to its anatomical position relative to the body’s center of mass [1,2]. Cephalad fluid redistribution can lead to cerebral venous congestion and increased intracranial pressure (ICP), potentially impairing cerebral autoregulation and altering cerebral blood flow (CBF) [3]. Astronauts on long-duration missions have reported multiple neurological symptoms, such as orthostatic intolerance and syncope, possibly resulting from inadequate cerebral perfusion. Moreover, accumulating evidence suggests that chronic cerebral hypoperfusion is associated with cognitive decline [4,5]. Maintaining optimal cognitive and behavioral performance is essential for the success of human space missions. Therefore, it is essential to quantify the extent of CBF alteration observed during long-duration and deep-space missions, as this provides a critical step toward safety in space exploration.

Three-dimensional pseudo-continuous arterial spin labeling (3D-pCASL) is currently the only noninvasive and nonradioactive magnetic resonance imaging (MRI) technique that enables absolute quantification of global and regional CBF [6]. Despite its advantages, the use of MRI in space stations is limited by its technical complexity and payload requirements. Consequently, real-time monitoring of CBF alteration during spaceflight remains a significant challenge. This underscores the importance of developing practical, lightweight, and astronaut-friendly approaches for CBF assessment, facilitating timely in-flight monitoring and protection of astronauts’ brain health.

Anatomically, the internal carotid artery (ICA) dominates the afferent blood flow to the cerebral circulation, serving as a primary conduit for blood supply to the brain [7]. Alterations in ICA blood flow have been shown to correlate closely with CBF, providing a physiological basis for predicting the cerebral perfusion status [8]. Crucially, astronauts can use portable ultrasound devices for ICA blood flow measurements in spaceflight [9].

**Fig. 1.**
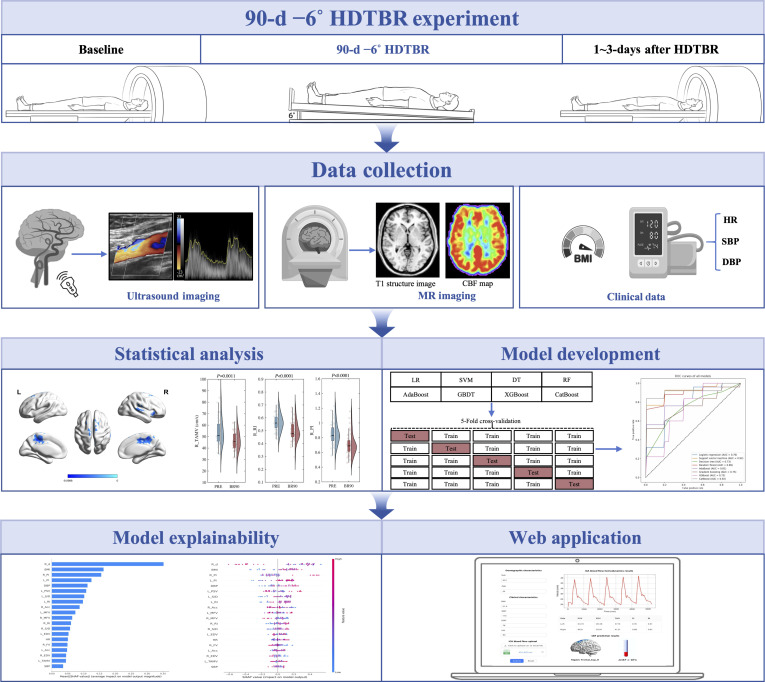
The schematic overview of the study design. First, multimodal imaging, demographic, and clinical data obtained from 90-d HDTBR experiment. Further statistical analyses were conducted for alteration of CBF. Next, the CBF prediction models were developed based on 8 ML methods and explanation analysis was utilized to identify the important input features. Finally, the prediction model was implemented as a web application.

The −6° head-down tilt bed rest (HDTBR) model has been widely utilized on Earth to replicate and simulate the physiological effects of microgravity in humans [10]. In this study, we conducted a prospective 90-d −6° HDTBR trial, during which ICA Doppler ultrasound and brain MRI data were collected. We first analyzed the whole brain CBF alteration induced by simulated microgravity and identified brain regions with significant CBF changes as predictive targets. Subsequently, the lightweight machine learning (ML) methods were used to establish the mapping relationship between carotid-brain blood flow in simulated microgravity for the CBF prediction. The explainability analysis was then conducted to assess feature importance and explain the final model’s results. Finally, an interactive web platform for the prediction model was developed for evaluating CBF, enabling astronauts to receive immediate guidance for countermeasures or medical interventions during space missions. Fig. 1 shows the schematic summary of the study design.

## Materials and Methods

### Study design and participants

This experiment was conducted as a part of the “Earth-Star II” 90-d −6° HDTBR project hosted by the China Astronaut Research and Training Center [11], during which 36 healthy male participants were exposed to 90-d strict −6° HDTBR. All participants underwent extensive physical and psychological screening. The exclusion criteria included contraindications for MRI, any diseases, and any cardiovascular, cerebrovascular, neurological, mental, or infectious diseases. The study was approved by the ethics committee of China Astronaut Research and Training Center (no. ACC201904) and was conducted in accordance with the tenets of the Declaration of Helsinki. Informed consent was obtained from all participants before enrolling the study.

During the HDTBR period, the participants strictly maintained a −6° HDT position without pillow, and they were required to have at least one shoulder touch the bed at all times (they can be supine, prone, or lateral) to ensure that a full-body −6° HDT angle was maintained. Daily activities included eating, drinking, showering, and toileting, performed in the HDT position on the bed, with 24/7 monitoring ensuring that they maintained their correct positions. Moreover, all participants ingested standardized isocaloric diets with controlled fluid intake and adhered to a strict sleep–wake schedule.

Multimodal imaging measurements were performed by trained physicians during two time points: (a) a 15-d baseline data collection period prior to HDTBR (denoted as PRE) and (b) within 1 to 3 d after the 90-d HDTBR period (denoted as BR90). Due to MRI devices and schedule constraints, the ultrasound and MRI scans were not conducted on the same day. In the PRE phase, the mean interval between the ultrasound and MRI scans was 5.25 d, whereas in the BR90 phase, the mean interval was 2 d. More details about this study design were summarized in the Supplementary Materials.

### Data collection and preprocessing

#### clinical data acquisition 

Heart rate (HR), systolic blood pressure (SBP), and diastolic blood pressure (DBP) were measured on the right brachial artery at heart level using an electronic sphygmomanometer (HEM-7133, Omron Corporation, Kyoto, Japan) after had maintained at least 5 min of rest without physical activity in the sitting position. Height and weight were measured in the morning, and the body mass index (BMI) was calculated as the weight in kilograms divided by the square of the height in meters.

#### Ultrasound imaging acquisition

Bilateral ICA B-mode and pulsed-wave Doppler ultrasound data were collected using a Mindray Resona7 ultrasound system (Mindray, Shenzhen, China). An L9-3U (3.0 to 9.0 MHz) high-frequency line array handheld probe was used. The parameters were set as follows: frame rate = 75 fps, dynamic range =120 dB, iClear = 5, iBeam = 1, Doppler angle ≤ 60°. The other parameters were individually adjusted. The pulsed Doppler sample volume was positioned at approximately 1.5 cm distal to the common carotid artery bifurcation. Participants were examined in the supine position. Two sonographers with at least 5 years of experience conducted all ultrasound examinations, and the sonographers were blinded to any specific information of the participants. Savitzky–Golay filtering and low-pass filter smoothing were used to eliminate noise and outliers from the Doppler data [12].

#### MRI acquisition

MRI data were acquired using a 3T Discovery MR750W MRI system (GE Healthcare, Waukesha, WI, USA) equipped with a 16-channel head-neck coil at PRE and BR90. Participants were instructed to keep their eyes closed and remain as still as possible during scanning, not to think of anything in particular, and to avoid falling asleep. Foam pads and earplugs were provided to the participants to minimize noise and head movement.

The ASL images were obtained used pseudo-continuous labeling, with background suppressed 3-dimensional stack-of-spiral fast spin echo readout trajectory: labeling duration = 1,450 ms, repetition time (TR) = 4,854 ms, echo time (TE) = 10.7 ms, post-labeling delay (PLD) = 2,000 ms, slice thickness = 4.0 mm, matrix size = 128 ×128, spiral-in readout = 512 × 8, field of view (FOV) = 240 mm × 240 mm, number of slices = 36, and scan duration = 4 min 34 s. The high-resolution 3-dimensional T1-weighted (T1w) images were obtained using 3D BRAVO for anatomical reference: TR = 8.8 ms, TE = 3.5 ms, flip angle (FA) = 15°, slice thickness = 1.0 mm, matrix size = 256 × 256, FOV = 240 mm × 240 mm, number of slices = 196, and scan duration = 4 min 36 s.

The MRI data were preprocessed using statistical parameter mapping software (SPM12) (https://www.fil.ion.ucl.ac.uk/spm) and the Data Processing and Analysis of Brain Imaging (DPABI) toolbox [13] implemented in MATLAB R2023a (The MathWorks Inc., Natick, MA, USA). The raw data of ASL were transferred to GE Advanced Functional Tools workstation to obtain the absolute CBF maps (mL/100 g/min). All images in DICOM format were converted into NIfTI format. First, the individual CBF images were co-registered to the corresponding T1w structural images and subsequently normalized into the Montreal Neurological Institute (MNI) space. After co-registration, the CBF maps were smoothed with 6-mm full width at half-maximum (FWHM) isotropic Gaussian kernel. Moreover, gray-matter (GM) and white-matter (WM) masks in MNI space were used to extract the absolute CBF values in GM and WM for region of interest (ROI)-wise analysis. For voxel-wise analysis, CBF maps were *z*-normalized by subtracting the global GM mean and dividing by the global GM standard deviation within the GM mask.

Quality control of all MR images was visually inspected by 2 independent and blinded neuroradiologists with 10 years of experience (H.W. and D.L.). The assessment focused on field-of-view coverage, contrast between GM and WM, and any other major artifact. The data considered to be of insufficient quality were excluded prior to analysis.

### Input features

The hemodynamic features of both the left and right ICA were considered including peak systolic flow velocity (PSV), end-diastolic flow velocity (EDV), mean blood flow velocity (MFV), time-averaged mean velocity (TAMV), resistance index (RI), pulsatility index (PI), systolic/diastolic ratio (*S*/*D*), acceleration (Acc), diameter (*d*), and flow volume (FV). Table S1 describes these features in detail. Additionally, the physiological features including age, BMI, HR, SBP, and DBP were considered. The above feature value differences (aside from age) between BR90 and PRE (${\text{feature value}}_{\mathrm{BR}90}-{\text{feature value}}_{\mathrm{PRE}}$) were used as inputs to the prediction model. To eliminate the influence of offset effects on the prediction results, the *z*-score method was used to normalize the input features.

### Definition of the outcome

The CBF is a measure of the volume of blood per unit time and is typically measured as perfusion in a given quantity of brain tissue (ml/100g/min) [14]. A sustained reduction in CBF can lead to irreversible neuronal injury, particularly in vulnerable brain regions. Accordingly, this study focuses on brain regions with significant reductions in absolute CBF and treats them as target regions for predicting CBF changes.

Although there is no universally established threshold for ischemic injury, it is estimated that a decrease in CBF to approximately 20% of baseline can result in cerebral ischemia within minutes, potentially leading to astronaut cognitive impairment [15–17]. Moreover, a previous study showed a 25.1% decrease in whole mean CBF at 7-d HDTBR and a 16.2% decrease at 29-d HDTBR [18]. We hypothesize that with increasing HDTBR duration, the magnitude of CBF changes decreases. In the context of spaceflight, early intervention is crucial to mitigating neurovascular risks. Based on guidance from clinical specialists, we adopted a 10% reduction in CBF as a precautionary threshold. This criterion serves as an early warning indicator, prompting timely countermeasures to protect astronaut brain health during extended space missions. Therefore, the prediction task can be regarded as a binary classification problem. A decrease in absolute CBF more than −10% in a brain region is considered warning, otherwise healthy. Formally, $\begin{array}{c} \left({\mathrm{CBF}}_{\mathrm{BR}90}-{\mathrm{CBF}}_{\mathrm{PRE}}\right)/{\mathrm{CBF}}_{\mathrm{PRE}}\times 100\%<\;-\;10\% \end{array}$ was considered to be a warning sign.

### Model development

Eight machine learning algorithms were used to construct the CBF prediction models: logistic regression (LR), support vector machine (SVM), decision tree (DT), random forest (RF), adaptive Boost (AdaBoost) [19], gradient boosting decision tree (GBDT) [20], extreme gradient boosting (XGBoost) [21], and Categorical Boosting (CatBoost) [22]. Given the imbalanced distribution of the dataset, the adaptive synthetic sampling (ADASYN) technique [23] was used to augment the original dataset, which generated new samples by synthesizing minority classes to obtain a balanced dataset. To prevent information leakage, ADASYN was fitted on the training fold only and the synthetic samples were generated within each training split. Model training and evaluation followed a stratified 5-fold cross-validation protocol to avoid overfitting. The evaluation process was repeated 10 times with varying random seeds in order to calculate the average metrics. A grid search algorithm was applied to select the optimal hyperparameters for each ML model (Table S2). 

Six main metrics were commonly considered to evaluate and compare the effectiveness of prediction models, that is, the accuracy (ACC), sensitivity (SEN), specificity (SPE), F1 score (F1), Matthews correlation coefficient (MCC), and area under the receiver operating characteristic curve (AUC) metrics. The models were optimized to achieve the best AUC as it is a threshold-independent performance measure that is familiar to clinicians.

Explanation is important for the prediction model when it is clinically applied. Finding key features that significantly contribute to results is an acritical mean to open the “black box” of ML. The model-agnostic method SHapley Additive exPlanations (SHAP) [24] method was used to analyze the significance and contribution of each feature in the output prediction based on the Shapley value. The Shapley value $\phi_{i}$ for each input feature is written as follows:

ϕi=∑S⊆N\iS!D−S−1!D!fxS∪i−fxS (1)

where N is the set of all input features, D is the dimension of all input features, S is the feature subset that excludes feature i, $f_{x}\left(S\cup \left\{i\right\}\right)$ is the model’s prediction using the features in S along with feature i, and $f_{x}\left(S\right)$ is the prediction using only the features in S.

### Statistical analysis

All statistical analyses were performed using SPSS version 26.0. ROI-wise analyses were conducted on absolute CBF (mL/100g/min). Voxel-wise analyses were performed on normalized CBF, and group differences were assessed using paired *t* tests with age included as a nuisance covariate. The Gaussian random field (GRF) method was used to correct for multiple comparisons, with significance set at voxel-level threshold at *P* < 0.001 and cluster-level threshold *P* < 0.01. The cluster extent threshold was set to greater than 500 voxels. The anatomical automatic labeling (AAL) brain atlas [25] was used to report the brain regions. The clinical characteristics and hemodynamic features between the 2 groups were compared using a 2-tailed paired *t* test. Spearman’s correlation was used to assess the association between percentage change in CBF and percentage change in clinical characteristics. The ML models were implemented using Python version 3.9.13 with the Scikit-learn, Pandas, and NumPy libraries. *P* < 0.05 was considered indicative of a statistically significant difference. Data are expressed as mean ± standard error of mean.

## Results

### Clinical characteristics

The 90-d strict −6° HDTBR internal cohort included 36 healthy male participants. The comparison focused on their clinical characteristics including height, weight, BMI, HR, SBP, and DBP (Table 1).

**Table 1.** Comparison of clinical characteristics in the HDTBR experiment

Characteristic	HDTBR (*n* = 36)		
	PRE	BR90	*P*
Age/years	32.0 ± 5.4	/	/
Height/m	167.7 ± 3.5	169.0 ± 3.5	<0.0001
Weight/kg	63.4 ± 6.7	62.5 ± 5.5	0.0825
BMI/(kg·m^−2^)	22.5 ± 2.4	21.9 ± 2.1	0.0009
HR/bpm	77.5 ± 11.3	65.7 ± 9.9	<0.0001
SBP/mmHg	117.3 ± 10.6	114.2 ± 9.1	0.1441
DBP/mmHg	71.9 ± 5.4	73.3 ± 7.0	0.2921

Compared to baseline (PRE), statistically significant changes in height (*P* < 0.0001), BMI (*P* = 0.0009), and HR (*P* < 0.0001) were observed after 90-d HDTBR (BR90). Notably, the SBP exhibited a decreasing trend, while the DBP exhibited an increasing trend, although the difference was not statistically significant.

### CBF decreased after HDTBR

All acquired ASL datasets passed the visual quality assessment and were included in the CBF analysis. There was no significant absolute CBF difference after 90-d HDTBR in whole brain [(47.40 ± 6.27) ml/100g/min versus (47.84 ± 7.12) ml/100g/min; *P* = 0.628], GM [(51.24 ± 6.94) ml/100g/min versus (51.44 ± 7.97) ml/100 g/min; *P* = 0.845], and WM [(42.48 ± 5.33) ml/100 g/min versus (42.83 ± 6.00) ml/100 g/min; *P* = 0.664].

**Fig. 2.**
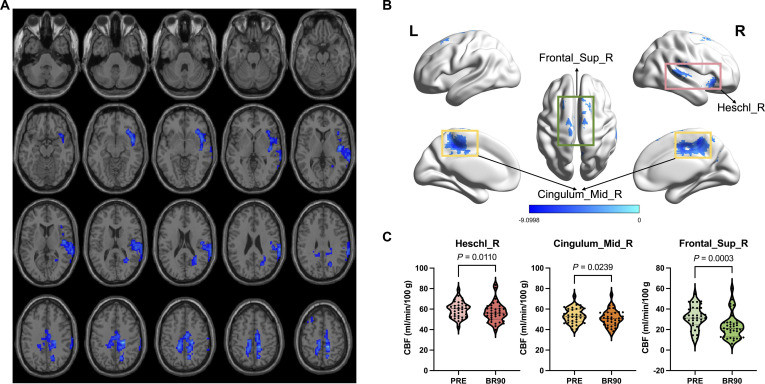
Group differences in CBF between PRE and BR90 at the voxel-wise group analysis (GRF corrected; cluster *P* < 0.01, voxel *P* < 0.001, 2-tailed). Clusters in blue represent decreased CBF after 90-d HDTBR. Significantly decreased CBF was observed in right Heschl’s gyrus (red box area, *P* = 0.0110), right middle cingulate gyrus (yellow box area, *P* = 0.0239), and right superior frontal gyrus (green box area, *P* = 0.0003). (A) Axial slices showing regions with significant decreases in CBF in BR90 compared to PRE. (B) 3D cortical surface images showing regions with significant decreases in CBF in BR90 compared to PRE. (C) Statistical results for brain regions with significantly decreased absolute CBF. L, left; R, right; Heschl_R, right Heschl’s gyrus; Cingulum_Mid_R, right middle cingulate gyrus; Frontal_Sup_R, right superior frontal gyrus.

Fig. 2A and B and Table S3 show locations and clusters with significant differences in the voxel-wise CBF, between the brain regions of the PRE and BR90 groups. Asymmetrical alterations of the CBF were observed, which significantly decreased in several brain regions including the right Heschl’s gyrus (Heschl_R; *x* = 45, *y* = −12, *z* = 6; *T* = −7.67), right middle cingulate gyrus (Cingulum_Mid_R; *x* = 18, *y* = −27, *z* = 42; *T* = −9.09), and right superior frontal gyrus (Frontal_Sup_R; *x* = 24, *y* = 12, *z* = 69; *T* = −5.5). Absolute CBF values were statistically analyzed for the voxel-wise significant clusters with decreased CBF, as shown in Fig. 2C. More details of the CBF changes are shown in Fig. S1.

Moreover, Table S4 showed that no significant association was observed between changes in CBF and changes in clinical characteristics.

### Alteration of ICA hemodynamic features

**Fig. 3:**
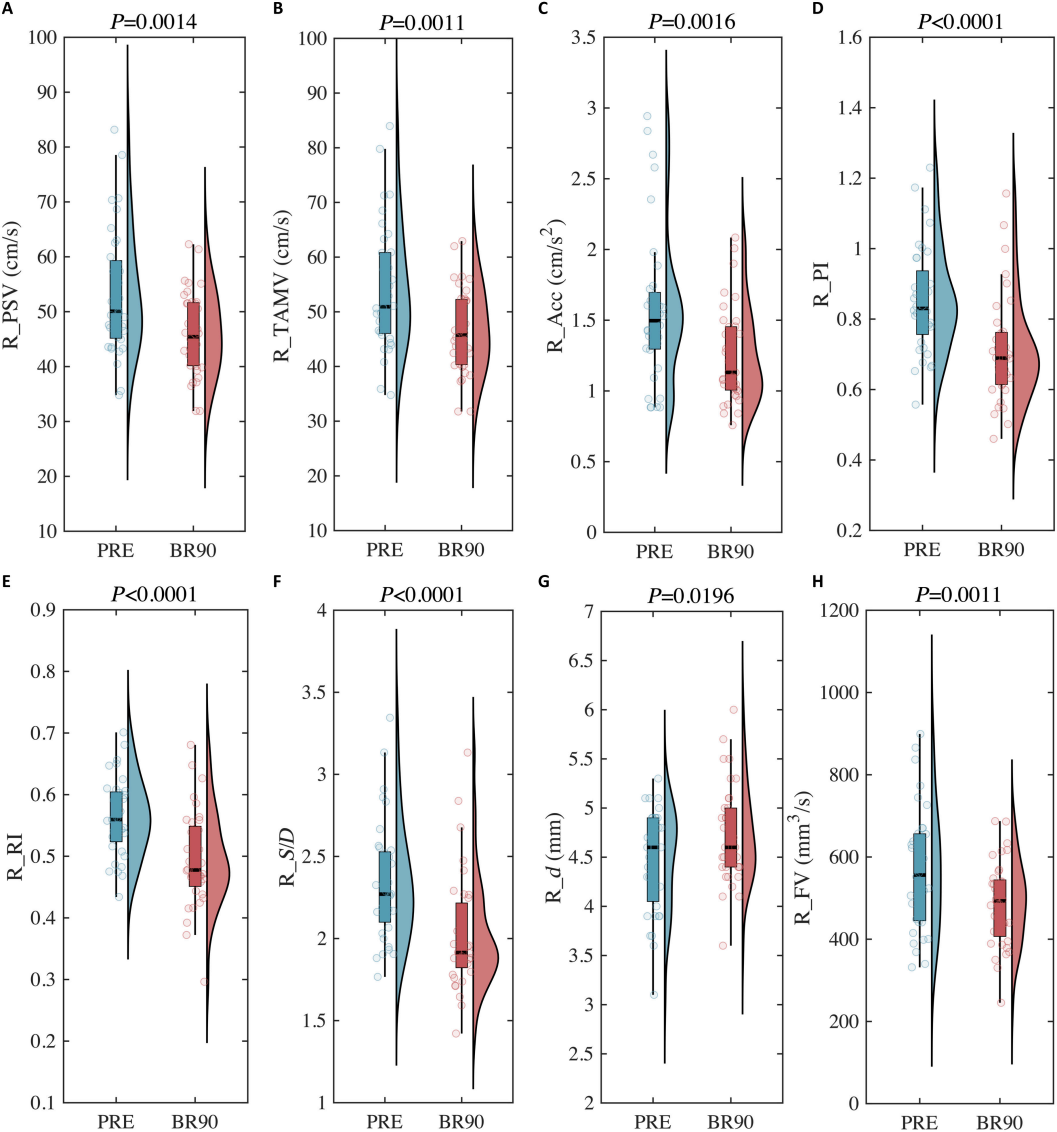
The alteration of hemodynamic features of right ICA in HDTBR experiment. (A) Difference in peak systolic velocity (PSV) of right ICA. (B) Difference in time-averaged maximum velocity (TAMV) of right ICA. (C) Difference in accelerated speed (Acc) of right ICA. (D) Difference in pulsatility index (PI) of right ICA. (E) Difference in resistance index (RI) of right ICA. (F) Difference in systolic/diastolic ratio (*S*/*D*) of right ICA. (G) Difference in diameter (*d*) of right ICA. (H) Difference in flow volume (FV) of right ICA.

The hemodynamic features of the right ICA exhibited significant differences before and after 90-d HDTBR. Specifically, R_PSV ((53.26 ± 1.88) cm/s versus (45.98 ± 1.26) cm/s, *P* = 0.0014), R_TAMV ((54.17 ± 1.91) cm/s versus (46.49 ± 1.28) cm/s, *P* = 0.0011), R_Acc ((1.577 ± 0.08) cm/s^2^ versus (1.243 ± 0.056) cm/s^2^, *P* = 0.0016), R_PI (0.85 ± 0.03 versus 0.71 ± 0.03, *P* < 0.0001), R_RI (0.56 ± 0.01 versus 0.49 ± 0.01, *P* < 0.0001), R_*S*/*D* (2.33 ± 0.06 versus 2.03 ± 0.05, *P* < 0.0001), and R_FV ((567.20 ± 24.67) mm^3^/s versus (486.50 ± 16.94) mm^3^/s, *P* = 0.0011) showed a significant decrease and R_*d* ((4.47 ± 10.08) mm versus (4.72 ± 10.08) mm, *P* = 0.0196) showed a significant increase after 90-d HDTBR (Fig. 3). Broadly speaking, there was a significant decrease in blood flow velocity, vascular resistance, and blood flow volume. The alteration of hemodynamic features in the left ICA (aside from L_*d*) was consistent with that of the right side (Fig. S2).

### Model prediction and performance comparison

Three region-specific CBF prediction models were developed for the right Heschl’s gyrus, right middle cingulate gyrus, and right superior frontal gyrus, which showed the greatest absolute CBF decrease. The comparative performance of 8 ML algorithms is presented in Table 2 and Fig. S3. Among them, the CatBoost algorithm exhibited the highest performance across all target brain regions (all AUCs were >0.80), indicating a remarkable advantage over the other algorithms. Specifically, for the CBF prediction performance in the middle cingulate gyrus, CatBoost achieved the highest AUC of 0.92, followed by SVM, RF, Adaboost, LR, GBDT, DT, and XGBoost. Additionally, the CatBoost model outperformed the others with accuracy of 0.84 [95% confidence interval (CI): 0.82 to 0.89] and F1 score of 0.85 (95% CI: 0.80 to 0.89), demonstrating robust performance across multiple evaluation metrics. Accordingly, the CatBoost algorithm was selected to construct the final CBF predictive model for all target brain regions.

**Table 2.** Performance of 8 ML models in predicting CBF among different brain regions. The 95% confidence intervals are included in parentheses.

Target regions	Model	ACC	SEN	SPE	F1	MCC
Heschl’s gyrus	LR	0.68 (0.63–0.72)	0.82 (0.71–0.89)	0.53 (0.40–0.62)	0.73 (0.62–0.73)	0.38 (0.34–0.42)
	SVM	0.71 (0.66–0.74)	0.92 (0.87– 0.95)	0.45 (0.42–0.49)	0.77 (0.74–0.79)	0.39 (0.37–0.42)
	DT	0.74 (0.71–0.79)	0.87 (0.85–0.90)	0.62 (0.58–0.64)	0.77 (0.74–0.81)	0.52 (0.47–0.57)
	RF	0.77 (0.72–0.82)	0.88 (0.85–0.94)	0.63 (0.58–0.66)	0.81 (0.77–0.85)	0.54 (0.51–0.58)
	Adaboost	0.74 (0.71–0.77)	0.85 (0.84–0.87)	0.58 (0.56–0.62)	0.77 (0.75–0.80)	0.45 (0.43–0.46)
	GBDT	0.73 (0.71–0.77)	0.82 (0.79–0.84)	0.67 (0.65–0.69)	0.76 (0.72–0.80)	0.52 (0.48–0.56)
	XGBoost	0.78 (0.73–0.83)	0.93 (0.90–0.95)	0.63 (0.60–0.67)	0.82 (0.78–0.87)	0.67 (0.63–0.69)
	CatBoost	0.84 (0.81–0.89)	0.93 (0.91–0.95)	0.73 (0.71–0.77)	0.87 (0.86–0.89)	0.58 (0.56–0.62)
Middle cingulate gyrus	LR	0.78 (0.70–0.82)	0.85 (0.77–0.89)	0.68 (0.64–0.73)	0.76 (0.67–0.79)	0.53 (0.49–0.55)
	SVM	0.78 (0.73–0.82)	0.97 (0.94–0.98)	0.62 (0.59–0.65)	0.82 (0.78–0.85)	0.61 (0.58–0.64)
	DT	0.70 (0.67–0.75)	0.78 (0.75–0.82)	0.60 (0.56–0.65)	0.73 (0.71–0.77)	0.43 (0.40–0.45)
	RF	0.78 (0.76–0.83)	0.88 (0.84–0.93)	0.70 (0.65–0.72)	0.79 (0.73– 0.83)	0.59 (0.55–0.66)
	Adaboost	0.62 (0.57–0.68)	0.78 (0.75–0.84)	0.45 (0.43–0.48)	0.69 (0.67–0.72)	0.23 (0.20–0.27)
	GBDT	0.74 (0.71–0.77)	0.80 (0.75–0.87)	0.68 (0.61–0.76)	0.73 (0.68–0.78)	0.53 (0.47–0.58)
	XGBoost	0.58 (0.52–0.66)	0.68 (0.63–0.74)	0.47 (0.42–0.51)	0.59 (0.55–0.64)	0.14 (0.12–0.17)
	CatBoost	0.84 (0.82–0.89)	0.97 (0.96–0.98)	0.68 (0.64–0.74)	0.85 (0.80–0.89)	0.68 (0.64–0.72)
Superior frontal gyrus	LR	0.46 (0.38–0.67)	0.47 (0.42–0.53)	0.43 (0.39–0.48)	0.43 (0.41–0.45)	0.11 (0.08–0.16)
	SVM	0.67 (0.59–0.76)	0.55 (0.48–0.60)	0.78 (0.73–0.80)	0.60 (0.56–0.65)	0.37 (0.33–0.45)
	DT	0.63 (0.56–0.68)	0.60 (0.55–0.64)	0.65 (0.63–0.69)	0.61 (0.55–0.68)	0.26 (0.23–0.30)
	RF	0.68 (0.65–0.73)	0.55 (0.53–0.59)	0.78 (0.75–0.83)	0.59 (0.53–0.65)	0.34 (0.30–0.39)
	Adaboost	0.54 (0.51–0.58)	0.33 (0.30–0.35)	0.75 (0.71–0.78)	0.40 (0.37–0.42)	0.08 (0.05–0.13)
	GBDT	0.67 (0.65–0.73)	0.58 (0.55–0.63)	0.78 (0.73–0.81)	0.54 (0.52–0.57)	0.39 (0.34–0.42)
	XGBoost	0.73 (0.70–0.75)	0.63 (0.61–0.66)	0.78 (0.72–0.82)	0.65 (0.61–0.69)	0.45 (0.41–0.48)
	CatBoost	0.73 (0.70–0.74)	0.78 (0.76–0.81)	0.67 (0.63–0.74)	0.72 (0.70–0.75)	0.46 (0.41–0.49)

### Model explanation

**Fig. 4.**
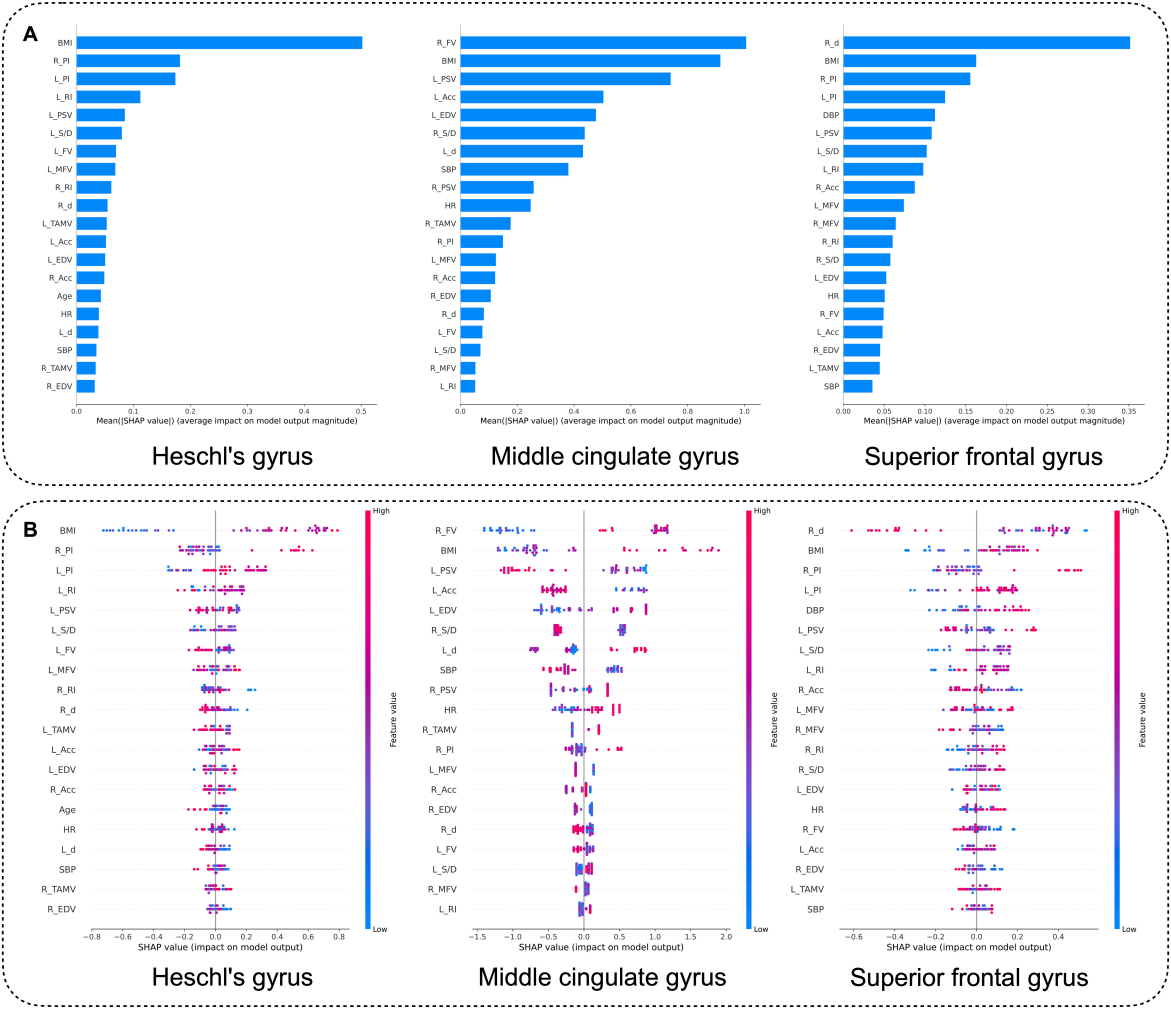
CBF prediction model explanation by the SHAP method. (A) SHAP summary bar plot. (B) SHAP summary dot plot. In (A), the *x* axis represents the average of the absolute SHAP values, indicating each feature’s contribution to the model’s predictions. In (B), the direction (color) and magnitude (position along the *x* axis) of each feature’s impact on the model prediction are displayed. Each point represents a sample, with color indicating the magnitude of the feature value (red for high values, blue for low values).

The SHAP method was utilized to interpret the output of the optimal model by calculating the contribution of each variable to CBF prediction. The relative importance of the clinical characteristics and hemodynamic features in each region-specific CBF prediction model is shown in Fig. 4. Notably, BMI was an important feature across all models. As illustrated in Fig. 4B, the larger the alteration in BMI, the more positively it contributed to the prediction results. For right Heschl’s gyrus, aside from the BMI, the features that impacted the CBF in descending levels of importance were as follows: PI of the right ICA (R_PI), PI of the left ICA (L_PI), and RI of the left ICA (L_RI). For the middle cingulate gyrus, the FV of the right ICA (R_FV) had a high and positive impact on model prediction, indicating that as the blood flow in ICA increased, the CBF decreased. Conversely, for the superior frontal gyrus, the larger the alterative value of the right ICA diameter, the more it contributed negatively to the model, implying that as the diameter expands, the probability of cerebral hypoperfusion was lower during simulated microgravity. Consistent with the right Heschl's gyrus prediction model, R_PI and L_PI also ranked among the influential features for the superior frontal gyrus.

### Web deployment

To facilitate the application to astronauts in spaceflight, we developed a web application using the Python Flask framework, designed to provide user-friendly, interactive, and explanatory visualization representations. Users can input demographic and clinical information (e.g., age, BMI, SBP, DBP, and HR) and upload ICA blood flow data for CBF prediction

As shown in Fig. S4, the web interface contained 3 modules: (a) input of subject information and clinical variables; (b) upload of ICA ultrasound file; and (c) visualization of prediction results, including alteration in ICA hemodynamics and the risk of warning CBF status.

## Discussion

In this study, we developed ML models to predict binary changes in CBF among individuals exposed to simulated microgravity. Our main findings are as follows: First, we observed that global CBF remained relatively stable, whereas regional CBF exhibited significant alterations, indicating spatially specific perfusion adaptations to simulated microgravity. Second, by applying various ML algorithms to model the relationship between carotid blood flow and CBF, we found that the CatBoost model achieved the superior performance, providing preliminary evidence that CBF can be inferred from in-orbit acquirable carotid ultrasound data. Third, BMI, vascular resistance, and blood flow volume of ICA emerged as key predictive features. To our knowledge, this is the first prospective study to employ multimodal image-driven ML for CBF prediction under long-duration simulated microgravity.

Previous studies have reported varying degrees of changes in CBF during spaceflight or simulated microgravity [26,27], partly because of individual differences, limited experimental sample sizes, and varying duration and conditions of experimental analogs. In this study, we firstly used 3D-pCASL technology to quantitatively assess global and regional CBF after 90-d HDTBR. The global CBF has good adaptability to simulated microgravity, and the body may gradually establish a new hemodynamic balance in the long-term simulated microgravity condition. By contrast, significant decreases were localized primarily to the right Heschl’s gyrus, right middle cingulate gyrus, and right superior frontal gyrus after 90-d HDTBR. These regions are critically involved in sensory integration, cognitive control, and executive function. Our finding revealed heterogeneous patterns of regional CBF change, potentially reflecting distinct neural adaptation strategies to long-duration simulated microgravity. In the absence of an internationally recognized microgravity-sensitive regions, our data-driven analysis identifies candidate regions that warrant heightened monitoring during spaceflight.

In addition, as the primary conduit for blood flow to the cerebral circulation, ICA has also been the focus of studies attempting to infer CBF from its flow characteristics. For example, Possnig et al. [28] reported a significant reduction in ICA blood flow volume after 3-d HDTBR, a finding consistent with our results. The mechanisms underlying decreases in CBF under microgravity remain incompletely understood but are likely multifactorial. One potential explanation is an elevation in ICP caused by cephalad fluid shift. In response, cerebral autoregulation acts to maintain stable CBF by dynamically adjusting vascular tone and cerebral vessel diameter [29]. In this study, we observed ICA dilation, accompanied by a reduction in blood flow velocity and cerebrovascular resistance. We speculate that these changes may reflect increased ICA compliance, which could contribute to the attenuation of pressure pulsatility induced by weightlessness. This hemodynamic adaptation may serve a protective role in maintaining CBF and mitigating potential adverse effects of ICP alterations in microgravity. However, further investigation is needed to distinguish between passive vascular changes and active autoregulatory mechanisms in prolonged microgravity exposure.

ML methods offer powerful means of extracting latent patterns from complex physiological datasets, enabling the modeling of nonlinear relationships between inputs and outputs. This capability has shown substantial promise in predicting physiological states. In this study, 8 ML algorithms were employed to predict CBF alterations under simulated microgravity conditions. Ensemble learning-based models, particularly GBDT, XGBoost, and CatBoost, demonstrated superior predictive performance compared with single ML methods (i.e., LR, SVM, and DT algorithms) across nearly all evaluation metrics. Notably, CatBoost consistently outperformed other approaches. As an advanced gradient boosting method, CatBoost integrates multiple individual learners, enabling it to capture complex interactions and patterns within the data more effectively.

We applied SHAP analysis to evaluate the contribution of each input feature to the final model’s predictions. The results indicate that BMI emerged as a key predictive feature across all prediction models, suggesting a potential link between change in BMI and change in CBF. Specifically, we observed a significant increase in height and a reduction in weight after 90-d HDTBR, resulting in a marked decrease in BMI. Indeed, the increased height was observed in both HDTBR or spaceflight experiments [30,31]. The most probable mechanism is swelling of the lumbar intervertebral disc in the unloaded condition during spaceflight. Moreover, the contributing factors to the decrease in weight could be that the distribution of fluid toward the head induces a reduction in antidiuretic hormone in the blood, leading to polyuria [32] or muscle loss following long-duration HDTBR [33]. Going further, previous studies on Earth have demonstrated that higher BMI is associated with reduced CBF [34–37]. One possible physiological mechanism is that increased blood flow to adipose tissue may divert cerebral blood supply, resulting in decreased CBF. Consistent with these findings, our data under simulated microgravity show a similar association direction. Additionally, we observed that decreased peripheral vascular resistance, reduced ICA blood flow volume, and dilation of the dominant ICA side under simulated microgravity were associated with alterations in CBF. While these vascular adaptations may contribute to changes in cerebral perfusion, their precise role in CBF regulation requires further investigation. It is important to emphasize that our findings indicate potential associations rather than causal relationships. While there is no established gold standard for ranking the relative importance of these vascular features, our findings are consistent with the radiologists and neurologists and are therefore unlikely to be regarded as controversial.

Despite these promising findings, our study had several limitations that warrant consideration. One notable limitation of our study is the lack of inclusion of the vertebral artery (VA) blood flow as the input feature of prediction model. The brain receives arterial perfusion through 2 principal arteries, and the mean relative contributions of ICA and VA to global CBF are 76% and 24%, respectively [38]. Integrating hemodynamic features of VA with ICA may improve the performance of the model. Nonetheless, Ogoh et al. [39] reported that the blood flow volume in VA was unchanged during and after the 3-d dry immersion (another microgravity simulation model). Future studies should further investigate the long-term effects of simulated microgravity on VA blood flow. Second, the single-PLD ASL protocol used in this study may fail to capture potential changes in arterial transit time and labeling efficiency arising from alterations in ICA diameter and flow velocity after 90-d HDTBR. Future work will adopt multi-PLD ASL acquisitions to characterize the inflow dynamics of labeled blood and mitigate labeling efficiency-related bias, ultimately improving the accuracy of CBF quantification. Third, only healthy male participants were recruited in this study. It remains unclear whether the proposed model will perform equally well in female. Given the increase in female astronauts in space missions, further research is needed to recruit diverse populations. Fourth, both the classification threshold and target prediction region were derived in a data-driven, population-level manner, which may introduce optimism bias; in the future, it will be necessary to explore astronaut-specific (personalized) prediction regions and thresholds to further improve predictive accuracy and clinical applicability. Fifth, to ensure dataset balance and minimize the influence of data partitioning on model training, we employed ADASYN technology and 5-fold cross-validation for data preprocessing. While these strategies help mitigate the risk of overfitting, the limited sample size and high feature dimensionality still constrain the model’s generalizability. Future research should validate the model’s performance in independent cohorts to confirm its robustness and utility. Finally, prior spaceflight experience, including the number of spaceflights and total spaceflight duration, leads to different CBF responses between first-time and frequent flyers. Future studies should record prior spaceflight history and incorporate it as a covariate or stratification factor in analysis when applied to astronaut populations.

In conclusion, this study elucidates the relationship between carotid blood flow and CBF under simulated microgravity and establishes lightweight, ML-based models capable of predicting CBF alterations. The proposed framework may serve as an early warning tool for neurovascular health monitoring in astronauts, thereby supporting long-duration human spaceflight.

## Data Availability

The data and codes are available on request from the corresponding author upon reasonable request.
