# Supplementary material for: Multimodal Imaging-Based Cerebral Blood Flow Prediction Model Development in Simulated Microgravity
Source: Cyborg Bionic Syst. 2025 Nov 24;6:0448. doi: 10.34133/cbsystems.0448 (PMC12641160; doi:10.34133/cbsystems.0448)
Supplement: Supplementary 1 — Supplementary Methods Figs. S1 to S4 Tables S1 to S4 [file cbsystems.0448.f1.docx]

Multimodal Imaging-Based Cerebral Blood Flow Prediction Model Development in Simulated Microgravity

**SUPPLEMENTARY MATERIALS**

# A.1 Details of HDTBR protocol

Inclusion criteria of this study were: (1) all physiological examination indicators within normal ranges, with no history of major illnesses; (2) no history of smoking or excessive alcohol consumption, and no recent use of haemodynamic-affecting medications; (3) absence of MRI contraindications (e.g., metallic implants, claustrophobia); (4) agreement to strictly adhere to the 90-day -6° HDTBR protocol. Exclusion criteria of this study were: (1) History of neurological disorders; (2) History of cardiovascular disease; (3) History of organic carotid artery disease; (4) History of chronic conditions or medication use affecting cerebral blood flow or vascular function; (5) History of allergies; (6) History of prolonged bed rest or participation in other microgravity simulation experiments; (7) Inability to adhere to the 90-day −6° HDTBR protocol and lifestyle management requirements throughout the study. Thirty-seven healthy male participants were recruited. However, one participant withdrew during adaptation period for personal reasons. Ultimately, thirty-six healthy male participants complete this study.

The HDTBR study comprised three phases: 15-day adaptation and baseline measurement period (ambulatory participants freely moving in the facility), 90 days of strict −6° HDTBR (without a pillow under the head), and a 33-day recovery phase. This study focused on the effects of simulated microgravity on CBF; therefore, recovery-phase results are not discussed in this paper.

During the 15-day pre-HDTBR period, participants were trained to perform all daily activities (eating, drinking, toileting, showering, urination, and defecation) in a −6° HDT position. Baseline physiological data were collected, and dietary assessments were conducted during this stage. Participants were ambulatory and slept in a horizontal position with ad libitum use of a normal pillow.

During the strict −6° 90-day HDTBR period, participants were not provided with pillows and were prohibited from raising their heads or propping themselves up. Their shoulders were required to remain in contact with the bed at all times, and all daily activities, including personal hygiene, were performed in the −6° head-down position. Specifically, participants ate and drank (using a straw) while lying face down, with no head elevation permitted during meals. Diet was strictly controlled by registered dietitians and provided by the clinical team, with identical composition for all participants. Two male nursing staff assisted in transferring participants to the toilet for defecation and showering. Urination was performed using a bedpan, while defecation was carried out on a specialized bed in the head-down position.

Participants were accommodated in double rooms separated by curtains, with ambient temperature maintained at 23–25 °C and standardized daily schedules (wake-up at 06:30, fixed mealtimes, and lights out at 23:00). Daytime napping was prohibited and monitored to ensure compliance. These measures were implemented to maintain circadian rhythm entrainment throughout the study. Bedside service devices provided in-bed entertainment, including movies and music. Continuous video surveillance and medical staff oversight ensured adherence to the bed rest protocol.

Nursing staff recorded daily physiological parameters, including blood pressure, heart rate, body temperature, fluid intake, and urine output. Body weight and height were measured weekly in the head-down position.

**Table S1**. The definition and Clinical implications of hemodynamic features in ICA blood flow.

| **Features** | **Definition** | **Clinical implications** |
| --- | --- | --- |
| $PSV cm/s$ | Maximum value of systolic blood flow velocity in the heart | Assessing the degree of arterial narrowing. |
| $\mathrm{EDV}cm/s$ | Blood flow velocity at the end of the diastolic phase of the heart | Assessing the severity of arterial stenosis. |
| $MFV cm/s$ | $MFV=\frac{1}{N}\sum_{i}^{N} V(t_{i})$  $V(t_{i})$ represents the blood flow velocity at each sampling point and $N$ is the total sampling points in a cardiac cycle. | Reflects mean blood flow throughout the cardiac cycle and is used for comprehensive assessment of blood flow. |
| $TAMV cm/s$ | $TAMV=\frac{1}{N}\sum_{i}^{N} V_{max}(t_{i})$  $V_{max}$ is the maximum velocity at each sampling point | Assessment of overall blood flow status, especially in evaluating long-term blood flow trends. |
| RI | $RI=\frac{\left( \mathrm{PSV}-\mathrm{EDV} \right)}{\mathrm{PSV}}$ | Elevated RI usually suggests increased downstream vascular resistance |
| PI | $PI=\frac{\left( \mathrm{PSV}-\mathrm{EDV} \right)}{\mathrm{MFV}}$ | Elevated PI usually suggests increased distal vascular resistance or decreased vascular elasticity |
| $S/D$ | $S/D=\frac{\mathrm{PSV}}{\mathrm{EDV}}$ | Elevated S/D ratio usually suggests increased distal resistance |
| $Acc \left( cm/s^{2} \right)$ | $AC=\frac{\left( \mathrm{PSV}-\mathrm{EDV} \right)}{t_{s}-t_{d}}$ | The growth rate of blood flow velocity from diastole to peak systole. |
| $d mm$ | measuring the ICA diameter on the B-mode image | Important indicators of local vascular lesions |
| Flow Volume (${mm}^{3}$/s) | $FV=\mathrm{TAMV}\times{(\frac{d}{2})}^{2}\times\pi\times60$ | Assessment of internal carotid artery perfusion |

The mean vessel diameter was first measured in the longitudinal cross-section using luminance mode (B mode). Blood flow was measured at least three times over a 2-minute period and the average values were used. $\mathrm{MFV}$ and $\mathrm{TAMV}$ were obtained from the average of multiple cardiac cycles to eliminate the effects caused by the breathing cycle.

**Table S2**. The optimized Hyperparameters.

| **Model** | **Hyperparameters** | **Search space** | **Optimal value** |
| --- | --- | --- | --- |
| LR | *C* | *{0.1,0.2,…,1.0}* | *0.01* |
|  | *penalty* | *{L1,L2, Elasticnet}* | *L2* |
|  | *solver* | *{newton-cg, lbfgs, liblinear, sag, saga}* | *liblinear* |
| SVM | *kernel* | *{Polynomial, Linear, rbf}* | *rbf* |
|  | *C* | *{0.1,1,10}* | *1* |
| DT | *max_ depth* | *{1,2,…,5}* | *2* |
|  | *min_sample_leaf* | *{2,3,…8}* | *2* |
|  | *min_sample_split* | *{1,2,3}* | *1* |
| RF | *n_estimators* | *{1,2,3,…,20}* | *8* |
|  | *min_sample_leaf* | *{1,2,3}* | *2* |
|  | *max_ depth* | *{1,2,…,5}* | *3* |
|  | *max_samples* | *{0.2,0.3,…,0.8}* | *0.5* |
|  | *boostrap* | *{True, False}* | *True* |
| AdaBoost | *n_estimators* | *{1,2,3,…,20}* | *6* |
|  | learning_rate | *[0.001, 0.01, 0.1, 0.2, 0.5, 1.0]* | *0.1* |
| GBDT | *n_estimators* | *{1,2,3,…,20}* | *3* |
|  | *min_sample_leaf* | *{1,2,3}* | *1* |
|  | *learning_rate* | *[0.001, 0.01, 0.1, 0.2, 0.5, 1.0]* | *0.001* |
| XGBOOST | *colsample_bytree* | *{0.8,0.9,1.0}* | *0.9* |
|  | *subsample* | *{0.5,0.8.1.0}* | *0.5* |
|  | *max_depth* | *{2,3,…10}* | *4* |
|  | *learning_rate* | *{0.01,0.02,…0.1,…,0.5}* | *0.001* |
|  | *min_child_weight* | *{2,3,…,10}* | *3* |
|  | *reg_alpha* | *{0,2,4,…,10}* | *0* |
| CatBoost | *iterations* | *{5,10,…50}* | *10* |
|  | *max_depth* | *{1,2,…,10}* | *3* |
|  | *learning_rate* | *{0.01,0.02,…,0.3}* | *0.02* |
|  | *leaf_estimation_method* | *{Newton, Gradient, Exact}* | *Newton* |
|  | *bootstrap_type* | *{Bayesian, Bernoulli, MVS}* | *MVS* |

**Table S3**. Significant clusters of voxel-wised comparisons in CBF between and after 90-day HDTBR. L, left; R, right.

| **Brain regions (AAL)** | **Peak MNI coordinates (mm)** | | | **Peak T value** | **Cluster size** |
| --- | --- | --- | --- | --- | --- |
|  | X | Y | Z |  |  |
| Heschl_R | 45 | −12 | 6 | −7.67625 | 1150 |
| Cingulum_Mid_R | 18 | −27 | 42 | −9.0998 | 1112 |
| Frontal_Sup_R | 24 | 12 | 69 | −5.53247 | 570 |

Peak coordinates of local maxima are reported per each cluster (maximum 3 maxima) in mm. Cluster size > 500 (k) are reported in voxels.

**Table S4**. The results of correlations between percentage changes in clinical characteristics and percentage changes in CBF in three target brain regions.

| **Clinical characteristics** | **Heschl_R** | | **Cingulum_Mid_R** | | **Frontal_Sup_R** | |
| --- | --- | --- | --- | --- | --- | --- |
|  | r | P value | r | P value | r | P value |
| Height | −0.012 | 0.943 | 0.067 | 0.696 | −0.155 | 0.366 |
| Weight | −0.134 | 0.437 | −0.126 | 0.465 | −0.319 | 0.058 |
| BMI | −0.129 | 0.452 | −0.114 | 0.507 | −0.245 | 0.150 |
| HR | −0.041 | 0.810 | −0.013 | 0.939 | −0.146 | 0.395 |
| SBP | −0.075 | 0.665 | −0.093 | 0.590 | 0.0545 | 0.752 |
| DBP | −0.221 | 0.195 | −0.281 | 0.096 | −0.112 | 0.517 |


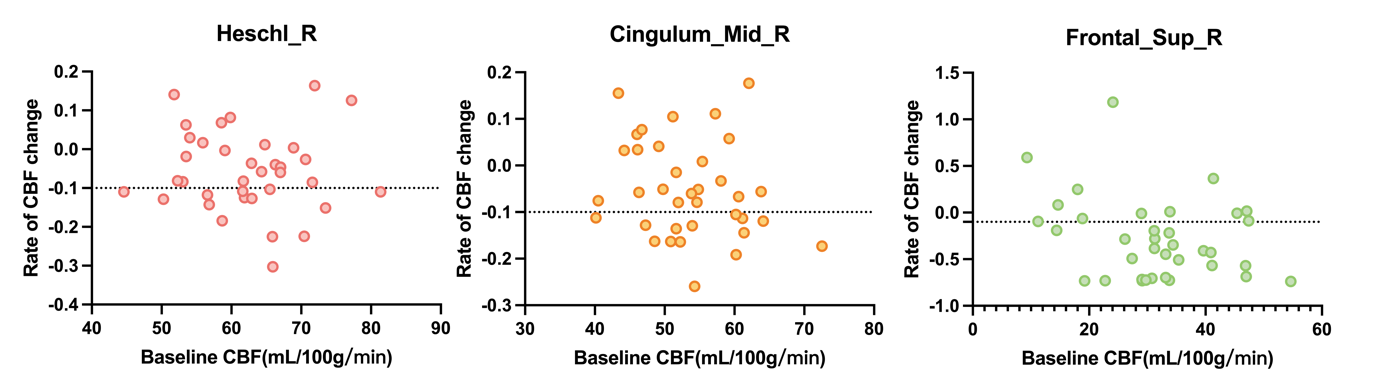


**Fig. S1.** Scatter plot of baseline CBF versus the rate of CBF change after 90-day HDTBR. Heschl_R, right Heschl’s gyrus; Cingulum_Mid_R, right middle cingulate gyrus; Frontal_Sup_R, right superior frontal gyrus.


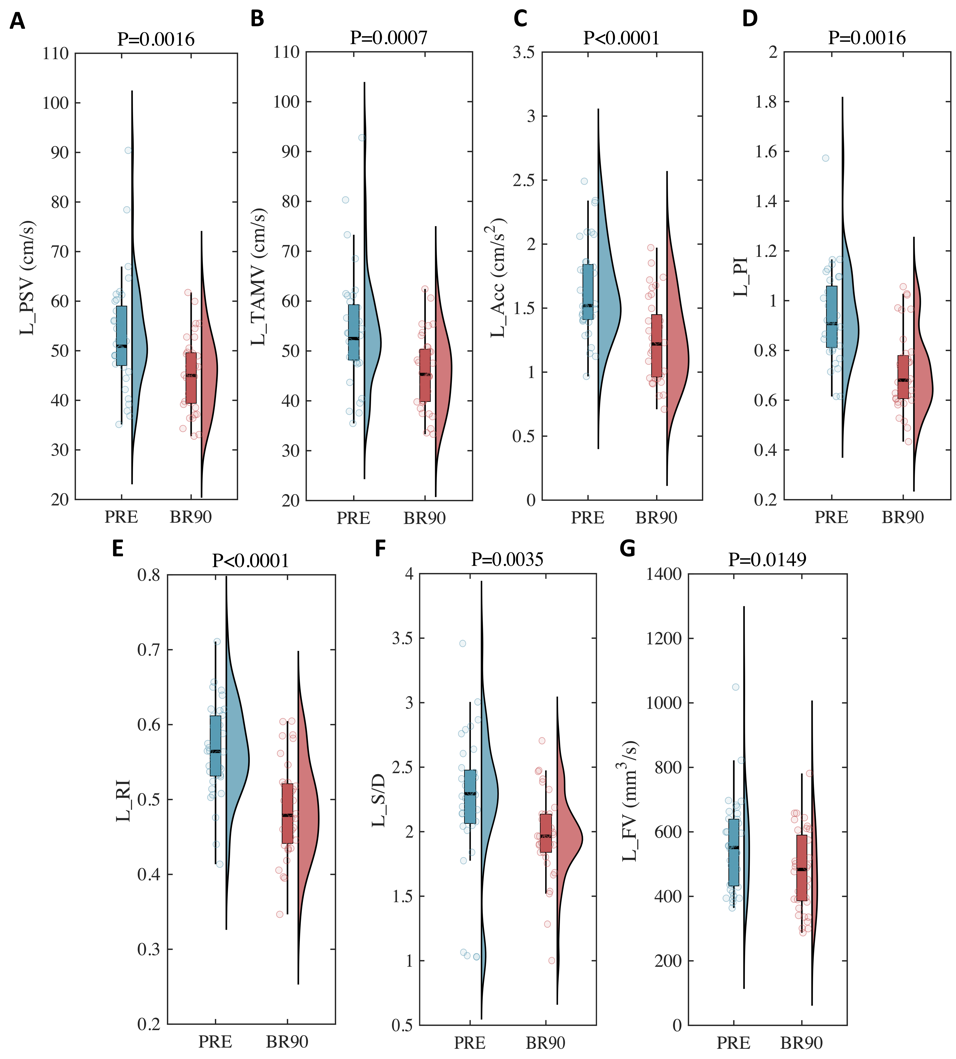


**Fig. S2.** The alteration of hemodynamic features of left ICA in HDTBR experiment. (A) The difference in PSV of left ICA. (B) The difference in TAMV of left ICA. (C) The difference in Acc of left ICA. (D) The difference in PI of left ICA. (E) The difference in RI of left ICA. (F) The difference in S/D of left ICA. (G) The difference in FV of left ICA. PSV, peak systolic velocity; TAMV, time averaged maximum velocity; Acc, accelerated speed; PI, pulsatility index; RI, resistance index; S/D, systolic/diastolic ratio; flow volume.


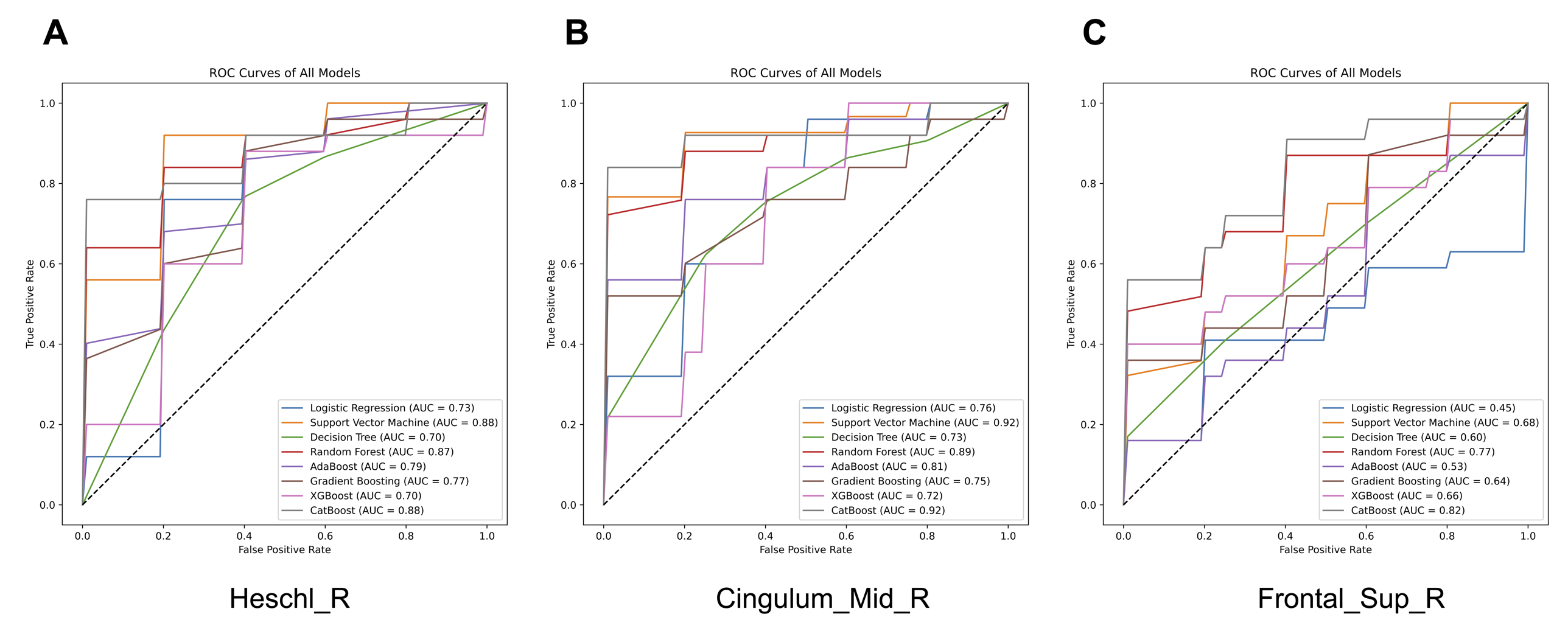


**Fig. S3**. The area under the receiver operating characteristic curve (ROC) for the eight ML models developed in the study. (A) ROC curve in Heschl’s gyrus (B) ROC curve in middle cingulate gyrus (C) ROC curve in superior frontal gyrus


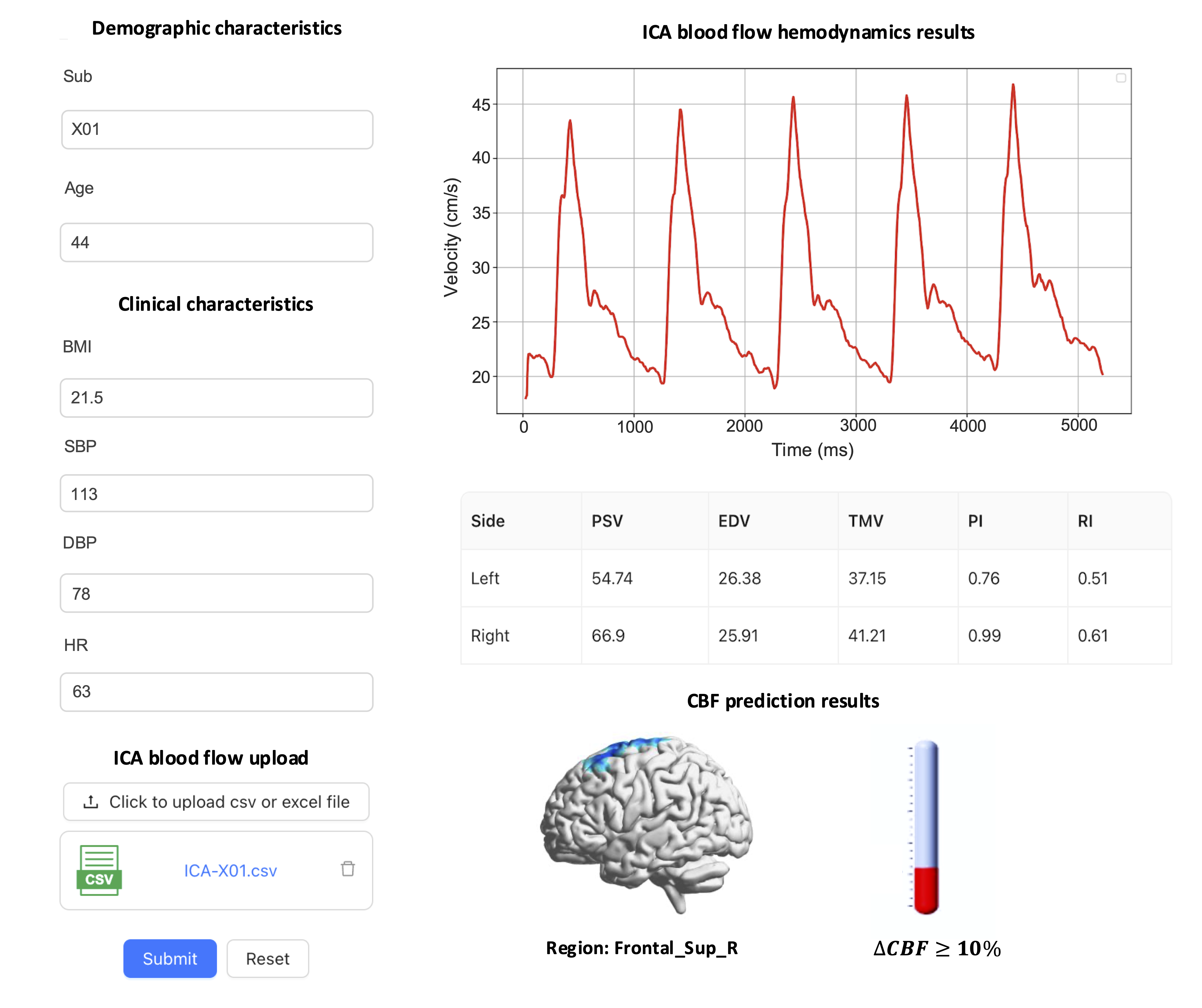


**Fig. S4.**  The user interface of the web application tool.
